# Long-term prognostic value of exercise technetium-99m tetrofosmin myocardial perfusion single-photon emission computed tomography

**DOI:** 10.1007/s12350-012-9585-y

**Published:** 2012-08-09

**Authors:** Hendrik J. Boiten, Johannes N. van der Sijde, Pauline R. Ruitinga, Roelf Valkema, Marcel L. Geleijnse, Eric J. G. Sijbrands, Ron T. van Domburg, Arend F. L. Schinkel

**Affiliations:** 1 Department of Cardiology, Thoraxcenter, Erasmus Medical Center, Rotterdam, The Netherlands; 2 Department of Nuclear Medicine, Erasmus Medical Center, Rotterdam, The Netherlands; 3 Department of Internal Medicine, Section of Pharmacology, Vascular and Metabolic Diseases, Erasmus Medical Center, Rotterdam, The Netherlands; 4 Department of Cardiology, Section of Pharmacology, Vascular and Metabolic Diseases, Thoraxcenter, Erasmus Medical Center, Room Ba304, ‘s-Gravendijkwal 230, 3015 CE Rotterdam, The Netherlands

**Keywords:** ^99m^Tc-Tetrofosmin, long-term prognosis, coronary artery disease, SPECT

## Abstract

**Background:**

Exercise ^99m^Tc-tetrofosmin single-photon emission computed tomography (SPECT) is a useful tool for short- and medium-term risk stratifications. Currently, the long-term prognostic application of this technique has not been evaluated.

**Methods and Results:**

Exercise ^99m^Tc-tetrofosmin was performed in 655 consecutive patients. Ten patients who underwent revascularization <60 days after nuclear testing were excluded from the analysis. The present data are based on 638 patients with complete follow-up. An abnormal SPECT study was defined as the presence of fixed and/or reversible perfusion defects. End points were cardiac death, nonfatal infarction, and late coronary revascularization. A total of 344 (54%) patients had an abnormal SPECT study. Perfusion defects included fixed defects alone in 186 patients (29%) and reversible defects in 158 (25%) patients. During a mean follow-up of 11.0 ± 3.3 years, 174 (27%) patients died (all-cause mortality). Nonfatal myocardial infarction occurred in 76 (12%) patients, and late coronary revascularization was performed in 194 (30%) patients. Univariable and multivariable Cox proportional hazard regression analyses showed that exercise ^99m^Tc-tetrofosmin SPECT provided prognostic information incremental to clinical data and exercise test data. Patients with a normal SPECT had a relatively favorable long-term prognosis, in contrast to patients with an abnormal study who had a significantly increased risk of cardiac events. The SPECT parameters abnormal scan, reversible defect, and summed rest score were strong predictors of long-term outcome.

**Conclusion:**

Exercise ^99m^Tc-tetrofosmin myocardial perfusion SPECT has an incremental long-term prognostic value over clinical and stress test parameters for the prediction of major adverse cardiac events.

## Introduction

Technetium-99m (^99m^Tc)-labeled agents are currently the most frequently used radionuclide tracers for the assessment of myocardial perfusion. Historically, ^99m^Tc-labeled agents were developed to overcome some limitations of thallium.^1^,^2^ These agents have a higher photon energy and shorter physical half-life, resulting in improved images quality and longer myocardial retention as compared to thallium.^3^ Multiple studies have demonstrated the value of ^99m^Tc-labeled tracers in conjunction with single-photon emission computed tomography (SPECT) for the diagnosis of coronary artery disease, and the estimation of medium-term prognosis.^3^-^10^ Several studies have demonstrated effective risk stratification with ^99m^Tc-tetrofosmin myocardial perfusion SPECT. Long-term outcome data after exercise ^99m^Tc-tetrofosmin myocardial perfusion SPECT are currently not available. Therefore, the aim of this study was to evaluate the long-term prognostic value of exercise ^99m^Tc-tetrofosmin SPECT for the prediction of major adverse cardiac events in patients with known or suspected coronary artery disease.

## Methods

### Study Design

The study population consisted of 655 consecutive patients, who underwent exercise ^99m^Tc-tetrofosmin SPECT imaging for the evaluation of known or suspected coronary artery disease. The current report details the results of a repeat follow-up from a prior study from our center.^6^ The reason to perform this repeat follow-up study was to assess the very long-term prognostic value of exercise ^99m^Tc-tetrofosmin myocardial perfusion SPECT. Diamond and Forrester clinical score was used to stratify patients with suspected coronary artery disease into groups with low, intermediate, or high probability of coronary artery disease. All patients gave informed consent before the test. The protocol was approved by the Hospital Ethics Committee. Ten patients who underwent revascularization <60 days after nuclear testing were excluded from the analysis. This exclusion was based on the previously published data indicating that referral to coronary revascularization in the first 60 days after nuclear testing tends to be based on the results of the scan and that referral to revascularization >60 days after nuclear testing tends to be based on the worsening of the patient’s clinical status.^11^ In December 2010, follow-up was performed. The present data are based on 638 patients with complete follow-up.

### Clinical Data

A structured interview and clinical history were obtained, and cardiac risk factors were assessed before nuclear testing. A blood pressure ≥140/90 mm Hg, or treatment with antihypertensive medication was considered as hypertension. A fasting glucose level ≥7.8 mmol/L or the need for insulin or oral hypoglycemic agents was considered as diabetes mellitus. A total cholesterol ≥6.4 mmol/L, or treatment with lipid-lowering medication was considered as hypercholesterolemia.

### Exercise Testing

All patients fulfilled a symptom-limited upright bicycle ergometry test with a stepwise increment of 20 W every minute and with three electrocardiographic leads continuously monitored. Cuff blood pressure measurements and twelve-lead electrocardiograms were recorded at rest and every minute during exercise and recovery. The Schiller system Cardiovit CSG/12 (Schiller Inc., Baar, Switzerland) was used for computer averaging of the electrocardiographic complexes. Significant ST-segment depression was defined as a >1-mm horizontal or downsloping ST-segment depression occurring at 80 ms after the J point.^6^ The target heart rate was defined as 85% of the maximum heart rate predicted for age and gender.

### ^99m^Tc-Tetrofosmin SPECT Imaging

An intravenous dose of 370 MBq of ^99m^Tc-tetrofosmin (Myoview, Amersham, Buckinghamshire, United Kingdom) was injected approximately 1 minute before the cessation of exercise. In rest studies, 370 MBq of tetrofosmin was administered at least 24 hours after the exercise study. Myocardial images were acquired with a triple-head gamma-camera system (Picker Prism 3000 XP, Cleveland, Ohio, USA). For each study, six oblique (short axis) slices from the apex to the base and three sagittal (vertical long axis) slices were defined. Each of the six short-axis slices was divided into eight equal segments. Owing to corresponding of the septal part of the two basal slices to the fibrous portion of the interventricular septum and normally exhibits reduced uptake, this region was excluded from analysis. As a consequence, 47 segments were indentified (3 long axis and 44 short axis). The interpretation of the scan was semiquantitatively performed by visual analysis and aided by circumferential profiles analysis. Exercise and rest tomographic views were reviewed side by side by an experienced observer who was blind to the patients’ clinical information. A reversible perfusion defect was defined as a perfusion defect on the exercise images that partially or completely resolved at rest in ≥2 contiguous segments or slices. A fixed perfusion defect was defined as a perfusion defect on exercise images in two or more contiguous segments or slices, which persists on rest images. The presence of a fixed and/or reversible perfusion defect was considered as an abnormal study. Each myocardial segment was assigned a score from 0 to 3 (0 = normal; 1 = slightly reduced; 2 = moderately reduced; and 3 = severely reduced or absent uptake). Summed stress score (SSS) and summed rest score (SRS) were calculated by the summation of the scores of the myocardial segments at stress and at rest, respectively. The difference between stress and rest scores, summed difference score (SDS), was considered representative of the extent and severity of myocardial ischemia. Standard 17-segment-based scores were calculated and converted into percent of the total myocardium (% myocardium) by dividing the summed scores by the maximum potential score, and multiplying by 100.^12^

### Follow-Up

Collection of follow-up data was performed by contacting the patient, the patient’s general practitioner, civil registries, and review of hospital records. Outcome events were overall mortality, cardiac death, nonfatal myocardial infarction, and late (>60 days) coronary revascularization. A death caused by acute myocardial infarction, significant arrhythmias, or refractory congestive heart failure was defined as cardiac death. Sudden death occurring without another explanation was included as cardiac death. Nonfatal myocardial infarction was described by cardiac biomarker levels and ECG-changes. Major adverse cardiac events were the combined endpoint of cardiac death, nonfatal myocardial infarction, or coronary revascularization.

### Statistical Analysis

Continuous variables were expressed as the mean ± SD and analyzed using the Student’s *t* test. The chi-squared test was used to compare categorical variables. Univariable and multivariable Cox proportional hazard regression models were performed to determine those variables which were independent predictors of late cardiac events. Variables were selected in a stepwise forward selection manner with entry and retention set of a significance level of 0.05. The risk of a variable was expressed as a hazard ratio (HR) with a corresponding 95% confidence interval. The assumption of the proportional hazards was evaluated by performing the log-minus-log survival plot. The assumption was met. Interaction terms were used to investigate collinearity; no interactions were found. The incremental value of myocardial perfusion SPECT over the clinical variables in the prediction of major adverse cardiac events was evaluated using a multivariate analysis including one pre-imaging model and three post-imaging models. Survival curves were generated using the Kaplan-Meier method to assess the probability of survival and were compared using the log-rank test. *P* < .05 was considered statistically significant.

## Results

### Clinical Characteristics and Exercise Test Results

The characteristics of the 638 patients are presented in Table 1. Diamond and Forrester pre-test probability of coronary artery disease was low in 51 (8%), intermediate in 364 (57%) and high in 223 (35%) patients. During the exercise test, typical angina was observed in 106 patients, and 124 patients showed significant ST-segment depression. Side effects were short ventricular tachycardia (<10 complexes) in, seven patients (1%), and atrial fibrillation in 8 (1%) patients. Minor side effects included dizziness in 40 (6%) patients, headache in 18 (3%) patients, and nausea in 12 (2%). Patients experienced no myocardial infarction or ventricular fibrillation.

**Table 1:** Baseline characteristics

n = 638	Number (%)
Age (years)	56 ± 11
Women	210 (33)
Congestive heart failure	70 (11)
Diabetes mellitus	58 (9)
Hypercholesterolemia	268 (42)
Hypertension	275 (43)
Previous myocardial infarction	171 (27)
Previous coronary artery bypass graft	91 (11)
Previous percutaneous coronary intervention	124 (19)
Known coronary artery disease	294 (46)
Smoking	153 (24)
Beta blockers	261 (41)
Calcium antagonists	269 (42)

### SPECT Results and Prognosis

**Figure 1:**
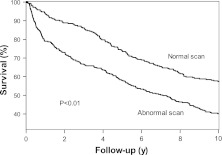
Kaplan-Meier survival curves for major adverse cardiac events (cardiac death, nonfatal infarction, and coronary revascularization). Event-free survival was significantly different in patients with a normal and patients with abnormal exercise ^99m^Tc-tetrofosmin myocardial perfusion SPECT. *y*, Years

**Figure 2:**
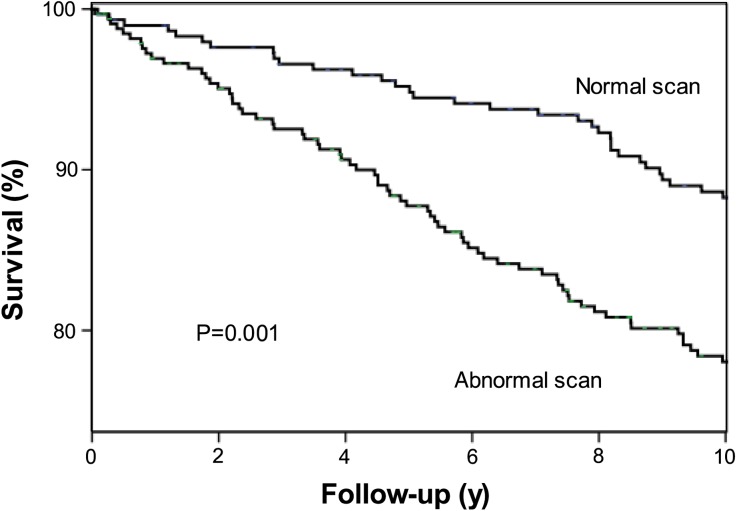
Kaplan-Meier survival curves for all-cause mortality. Event-free survival was significantly different in patients with a normal and patients with abnormal exercise ^99m^Tc-tetrofosmin myocardial perfusion SPECT. *y*, Years

**Figure 3:**
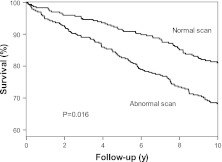
Kaplan-Meier survival curves for all-cause mortality and nonfatal myocardial infarction. Event-free survival was significantly different in patients with a normal and patients with abnormal exercise ^99m^Tc-tetrofosmin myocardial perfusion SPECT. *y*, Years

**Figure 4:**
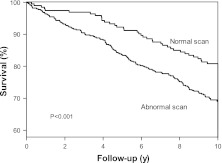
Kaplan-Meier survival curves for revascularization. Event-free survival was significantly different in patients with a normal and patients with abnormal exercise ^99m^Tc-tetrofosmin myocardial perfusion SPECT. *y*, Years

**Figure 5:**
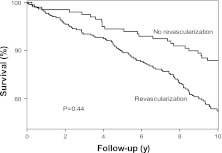
Kaplan-Meier survival curves for the combined endpoint of all-cause mortality or nonfatal myocardial infarction according to strata of coronary revascularization. Event-free survival was not significantly different between patients with and those without revascularization during follow-up. *y*, Years

A total of 344 (54%) patients had an abnormal SPECT study. Perfusion defects included fixed defects alone in 186 patients (29%) and reversible defects in 158 (25%) patients. Of these reversible defects, 56 were completely reversible and 102 were partially reversible. The means (SD) of the SPECT parameters SSS, SRS, and SDS were 3.68 (2.86), 2.43 (2.55), and 1.25 (1.30), respectively. During a mean follow-up of 11.0 ± 3.3 years, 174 (27%) patients died (all-cause mortality). Nonfatal myocardial infarction occurred in 76 (12%) patients, and late coronary revascularization was performed in 194 (30%) patients. The Kaplan-Meier survival curves are presented in Figures 1, 2, 3, 4, and 5. The survival curves show that a normal ^99m^Tc-tetrofosmin myocardial perfusion SPECT was associated with relatively low risk for major adverse cardiac events. Conversely, patients with an abnormal study had a significantly increased risk of major adverse cardiac events. Figure 5 demonstrates survival curves for all-cause mortality and nonfatal myocardial infarction according to strata of coronary revascularization.

### Predictors of Outcome

Predictors of major adverse cardiac events are demonstrated in Table 2. Univariable predictors of major adverse cardiac events were age, male gender, hypercholesterolemia, previous myocardial infarction, revascularization, peak heart rate, rate-pressure product, typical angina and all analyzed scan parameters. A multivariable model revealed that myocardial perfusion SPECT had an incremental prognostic value over clinical variables and stress test parameters. The post-imaging models demonstrated that an abnormal scan was a powerful predictor of outcome; there was a direct relation between an abnormal scan and the risk of major adverse cardiac events.

**Table 2:** Predictors of major adverse cardiac events (cardiac death, nonfatal infarction, or revascularization) at univariable and multivariable analyses

	Univariable analysis	Multivariable analysis			
		Pre-imaging	Post-imaging I	Post-imaging II	Post-imaging III
Age*	1.02 (1.01–1.03)	1.05 (1.03–1.06)	1.03 (1.01–1.04)	1.02 (1.01–1.03)	1.05 (1.03–1.08)
Male gender	1.89 (1.50–2.39)	1.80 (1.29–2.51)	*P* = .11	1.40 (1.09–1.79)	1.97 (1.25–3.10)
Congestive heart failure	*P* = .69	*P* = .62	*P* = .65	*P* = .78	*P* = .15
Diabetes mellitus	*P* = .22	1.96 (1.26–3.08)	1.74 (1.13–2.69)	*P* = .13	2.36 (1.35–4.14)
History of angina	*P* = .26	*P* = .63	*P* = .93	*P* = .66	*P* = .52
Hypercholesterolemia	1.30 (1.06–1.60)	*P* = .79	*P* = .89	*P* = .17	*P* = .92
Hypertension	*P* = .58	*P* = .82	*P* = .87	*P* = .10	*P* = .80
Previous infarction	1.58 (1.26–1.97)	1.71 (1.15–2.55)	1.51 (1.10–2.08)	1.33 (1.06–1.68)	*P* = .06
Revascularization	4.54 (3.63–5.68)	1.93 (1.43–2.60)	3.23 (2.43–4.30)	3.64 (2.93–4.53)	3.11 (2.32–4.16)
Smoking	*P* = .16	2.25 (1.50–3.38)	1.42 (1.03–1.94)	*P* = .18	2.41 (1.60–3.64)
Exercise test results					
Peak heart rate	0.87 (0.83–0.91)	*P* = .16	*P* = .31	0.98 (0.97–0.99)	*P* = .21
Peak systolic BP	*P* = .91	*P* = .29	*P* = .16	*P* = .86	*P* = .11
Peak RPP	0.96 (0.95–0.98)	*P* = .70	*P* = .94	*P* = .56	*P* = .93
Typical angina	1.68 (1.30–2.18)	*P* = .40	*P* = .53	*P* = .94	*P* = .60
ST-segment changes	*P* = .12	*P* = .10	*P* = .33	*P* = .20	*P* = .41
Scan parameters					
Abnormal scan	1.68 (1.36–2.08)	–	1.57 (1.12–2.20)	–	–
Reversible defect	1.94 (1.55–2.44)	–	–	1.62 (1.20–2.20)	–
Fixed defect	1.28 (1.03–1.58)	–	–	*P* = .47	
SRS**	1.02 (1.01–1.02)	–	–	–	1.03 (1.01–1.04)
SDS**	1.05 (1.04–1.07)	–	–	–	*P* = *.49*
SSS**	1.27 (1.15–1.46)	–	–	–	–

Statistically significant predictors of outcome are presented as hazard ratio (confidence interval), of all other variables the *P* value is presented.

–, Not included in the model; *BP*, blood pressure; *RPP*, rate pressure product; *SSS*, summed stress score; *SRS*, summed rest score; *SDS*, summed difference score.

* Per unit increment, ** per % myocardium increment.

## Discussion

The prognostic value of exercise ^99m^Tc-tetrofosmin myocardial perfusion SPECT has been reported in previous studies with short-to-medium-term follow-up.^4^-^10^ The long-term prognostic value of exercise ^99m^Tc-tetrofosmin myocardial perfusion SPECT has not been defined. This study demonstrates that the prognostic value of exercise ^99m^Tc-tetrofosmin myocardial perfusion SPECT in predicting major adverse cardiac events, all-cause mortality, and all-cause mortality and nonfatal myocardial infarction was maintained during a long-term follow-up of 11.0 ± 3.3 years. Univariable and multivariable analyses showed that exercise ^99m^Tc-tetrofosmin SPECT provided prognostic information incremental to clinical data and exercise test data. Patients with a normal SPECT had a relatively favorable long-term prognosis, in contrast to patients with an abnormal study who had a significantly increased risk of cardiac events. The SPECT parameters abnormal scan, reversible defect, and SRS were strong predictors of long-term outcome.

### Comparison to Previous Studies

Previous studies have demonstrated the prognostic value of exercise ^99m^Tc-tetrofosmin myocardial perfusion SPECT for the prediction of cardiac events in patients with known or suspected coronary artery disease at short-medium term follow-up.^4^-^10^ Groutars et al^4^ studied 246 patients who had a normal exercise thallium/^99m^Tc-tetrofosmin myocardial perfusion SPECT. The annualized cardiac event rate was 0.4%/year during a 25 ± 3 month follow-up. Galassi et al^5^ studied 459 patients with exercise ^99m^Tc-tetrofosmin myocardial perfusion SPECT. During a median follow-up of 38 months, the annualized event rate (cardiac death/nonfatal infarction) was 0.5%/year in patients with a normal study and 4.9%/year in those with an abnormal study. Shaw et al^7^ reported the results from a multicenter registry and described an excellent prognosis for patients with a normal ^99m^Tc-tetrofosmin myocardial perfusion SPECT during a mean follow-up of 2.5 years (30 months). Borges-Neto et al^8^ studied 473 patients with ^99m^Tc-tetrofosmin and 518 patients using ^99m^Tc-sestamibi exercise myocardial perfusion SPECT. The study demonstrated that the type of ^99m^Tc-labeled myocardial perfusion agent did not affect interpretation of results for risk stratification and prognostic assessment during the 1.5-year study period. Georgoulias et al^9^ studied 246 asymptomatic patients after percutaneous coronary intervention with exercise ^99m^Tc-tetrofosmin gated-SPECT. During 8.3 ± 2.9-year follow-up of this selected population, myocardial perfusion SPECT provided incremental prognostic value. However, routine testing in asymptomatic patients is generally not recommended, and the clinical value and cost-effectiveness of routine SPECT after percutaneous coronary intervention need further investigation. Recently, Jain et al^10^ studied 371 patients with exercise or pharmacologic stress ^99m^Tc-tetrofosmin gated-SPECT. During a mean follow-up of 3.9 years, SPECT findings significantly improved accuracy of cardiac event rate prediction compared to clinical information alone.

Compared to these previous studies, our study included 638 patients who were followed for 11.0 ± 3.3 years. The findings of the current study extend the conclusions drawn from the previous medium-term prognostic studies. Previously, we have reported the 4.0 ± 1.3-year follow-up of these 655 patients who underwent exercise ^99m^Tc-tetrofosmin myocardial perfusion SPECT.^6^ In the same previous study, patients with a normal study had an annualized event rate (cardiac death, nonfatal infarction, and coronary revascularization) of 1.5%/year and those with an abnormal study of 5.0%/year. In the current long-term follow-up study, Kaplan-Meier survival curves continued to diverge over time. Patients with normal SPECT had a favorable event-free survival, indicating that the prognostic value of SPECT for the prediction of outcome was maintained during the long-term follow-up period. Hence, patients with normal SPECT scans can be excluded from coronary invasive procedures. In contrast, patients with an abnormal myocardial perfusion SPECT had significantly higher annualized event rates. A total of 294 (46%) patients had known coronary artery disease; this may have caused the relatively high event rate in this patient cohort.

Future studies are needed to optimize risk modification to improve outcome in these patients.

The 10 excluded patients who underwent revascularization <60 days after testing represent 1.6% of the overall cohort. This percentage is relatively low because in most of the patients with ischemia according to exercise ^99m^Tc-tetrofosmin, medical therapy was probably optimized before a decision on referral to percutaneous coronary revascularization or surgical coronary revascularization was made. Kaplan-Meier curves demonstrated that referral to coronary revascularization was relatively low in patients with normal scan results in the first 4 years after testing. Previous studies demonstrate that the timing of revascularization requires careful consideration.^13^ Patients with no or mild symptoms and little ischemia can safely be treated with medical treatment alone. Conversely, patients with moderate-to-severe symptoms and/or extensive ischemia should be strongly considered for revascularization therapy.^13^ The current analysis demonstrates that event-free survival from all-cause mortality and nonfatal myocardial infarction was not significantly different between patients with coronary revascularization during follow-up and those without. This is in line with results from the COURAGE trial which demonstrated that clinical outcome was not significantly different between patients with stable angina who received an initial therapy of coronary revascularization and optimal medical therapy compared with patients with optimal medical therapy alone.^14^

### Limitations

This study has several limitations. First, no attenuation or scatter correction was used during exercise ^99m^Tc-tetrofosmin myocardial perfusion SPECT. Application of attenuation or scatter correction may have improved the accuracy of the SPECT studies. Recent data indicate that attenuation correction may further improve risk stratification.^15^,^16^ Second, previous studies have demonstrated that functional data derived from gated myocardial perfusion SPECT provides additional information to predict outcome. At the time of data collection, gated SPECT was not routinely performed. Therefore, in this study, the prognostic value of gated SPECT was not analyzed. Third, coronary angiography was not routinely performed; therefore, the current analysis may be influenced by false-positive and false-negative SPECT studies. Fourth, although the feasibility of the test was high, 72 (11%) patients had an inconclusive test (failure to achieve target heart rate and to demonstrate a perfusion abnormality). These 72 studies were considered normal. The feasibility and perhaps the prognostic value could have been higher if beta-blocker therapy was routinely discontinued before the exercise test. Fifth, the results of exercise ^99m^Tc-tetrofosmin myocardial perfusion SPECT were available to the treating physicians. Patient management decisions were made at discretion of the treating physicians. It cannot be excluded that patients with an abnormal study received more intensive medical therapy and were referred to coronary revascularization to favorably alter their prognosis. Therefore the prognostic power of exercise ^99m^Tc-tetrofosmin myocardial perfusion SPECT may have been underestimated in the present study. Sixth, the present results were obtained in patients who underwent exercise ^99m^Tc-tetrofosmin imaging, and cannot be automatically be extrapolated to SPECT studies using other isotopes. Finally, clinical data upon which to adjust the multivariable model were relatively limited; this could have influenced the current analysis.

## Conclusion

Exercise ^99m^Tc-tetrofosmin myocardial perfusion SPECT has an incremental long-term prognostic value over clinical and stress test parameters for the prediction of major adverse cardiac events. Patients with a normal ^99m^Tc-tetrofosmin SPECT have a relatively favorable long-term prognosis, in contrast to patients with an abnormal study who have a significantly increased risk of cardiac events.

## References

[1] Higley B, Smith FW, Smith T (1993). Technetium-99m-1,2-bis[bis(2-ethoxyethyl)phosphino]ethane: Human biodistribution, dosimetry and safety of a new myocardial perfusion imaging agent. J Nucl Med.

[2] Jain D, Wackers FJ, Mattera J, McMahon M, Sinusas AJ, Zaret BL (1993). Biokinetics of technetium-99m-tetrofosmin: Myocardial perfusion imaging agent: Implications for a one-day imaging protocol. J Nucl Med.

[3] Zaret BL, Rigo P, Wackers FJ (1995). Myocardial perfusion imaging with 99mTc tetrofosmin. Comparison to 201Tl imaging and coronary angiography in a phase III multicenter trial. Tetrofosmin International Trial Study Group. Circulation.

[4] Groutars RGEJ, Verzijlbergen JF, Muller AJ (2000). Prognostic value and quality of life in patients with normal rest thallium-201/stress technetium 99m-tetrofosmin dual-isotope myocardial SPECT. J Nucl Cardiol.

[5] Galassi AR, Azzarelli S, Tomaselli A (2001). Incremental prognostic value of technetium-99m-tetrofosmin exercise myocardial perfusion imaging for predicting outcomes in patients with suspected or known coronary artery disease. Am J Cardiol.

[6] Schinkel AFL, Elhendy A, Van Domburg RT (2003). Incremental value of exercise technetium-99m tetrofosmin myocardial perfusion single-photon emission computed tomography for the prediction of cardiac events. Am J Cardiol.

[7] Shaw LJ, Hendel R, Borges-Neto S (2003). Prognostic value of normal exercise and adenosine 99mTc-tetrofosmin SPECT imaging: Results from the multicenter registry of 4,728 patients. J Nucl Med.

[8] Borges-Neto S, Tuttle RH (2004). Outcome prediction in patients at high risk for coronary artery disease: Comparison between 99mTc tetrofosmin and 99mTc sestamibi. Radiology.

[9] Georgoulias P, Tzavara C, Demakopoulos N (2008). Incremental prognostic value of (99m)Tc-tetrofosmin myocardial SPECT after percutaneous coronary intervention. Ann Nucl Med.

[10] Jain D, Lessig H, Patel R (2009). Influence of 99mTc-tetrofosmin SPECT myocardial perfusion imaging on the prediction of future adverse cardiac events. J Nucl Cardiol.

[11] Hachamovitch R, Berman DS, Kiat H (1996). Exercise myocardial perfusion SPECT in patients without known coronary artery disease: Incremental prognostic value and use in risk stratification. Circulation.

[12] Hachamovitch R, Hayes S, Friedman JD (2003). Comparison of the short-term survival benefit associated with revascularization compared with medical therapy in patients with no prior coronary artery disease undergoing stress myocardial perfusion single photon emission computed tomography. Circulation.

[13] Simoons ML, Windecker S (2010). Controversies in cardiovascular medicine: Chronic stable coronary artery disease: Drugs vs. revascularization. Eur Heart J.

[14] Boden WE, O’Rourke RA, Teo KK (2007). Optimal medical therapy with or without PCI for stable coronary disease. N Engl J Med.

[15] Baghdasarian SB, Noble GL, Ahlberg AW (2009). Risk stratification with attenuation corrected stress Tc-99m sestamibi SPECT myocardial perfusion imaging in the absence of ECG-gating due to arrhythmias. J Nucl Cardiol.

[16] Pazhenkottil AP, Ghadri JR, Nkoulou RN (2011). Improved outcome prediction by SPECT myocardial perfusion imaging after CT attenuation correction. J Nucl Med.

